# Nutritional Status, Body Composition and Cardiometabolic Profile in Individuals with Tetraplegia: A Pilot Cross-Sectional Study

**DOI:** 10.3390/medicina61122182

**Published:** 2025-12-08

**Authors:** María Martínez-Olcina, Ángel Camblor-Navarro, Bernardo José Cuestas-Calero, Yolanda Nadal-Nicolás, Belén Leyva-Vela, Manuel Vicente-Martínez, Izan Rodríguez-López, Rodrigo Yáñez-Sepúlveda, Guillermo Cortés-Roco, Alejandro Martínez-Rodríguez, Aarón Manzanares

**Affiliations:** 1 Departamento Química Analítica, Nutrición y Bromatología, Universidad de Alicante, 03690 Alicante, Spain; maria.martinezolcina@ua.es (M.M.-O.); bmlv1@alu.ua.es (B.L.-V.); mvmr2@alu.ua.es (M.V.-M.); irl26@alu.ua.es (I.R.-L.); amartinezrodriguez@ua.es (A.M.-R.); 2 Facultad de Deporte, UCAM Universidad Católica de Murcia, 30107 Murcia, Spain; camblornavarro10@gmail.com; 3 Faculty of Health Sciences, Universidad Europea de Valencia, 46010 Valencia, Spain; bernardojose.cuestas@universidadeuropea.es; 4 Department of Pathology and Surgery, Faculty of Medicine, Miguel Hernández University of Elche, 03202 Elche, Spain; yolanda.nadal@umh.es; 5 Facultad de Educación y Ciencias Sociales, Instituto del Deporte y Bienestar, Universidad Andres Bello, Las Condes, Santiago 7550000, Chile; rodrigo.yanez.s@unab.cl; 6 School of Medicine, Universidad Espíritu Santo (UEES), Samborondón 091650, Ecuador; 7 School of Education, Pedagogy in Physical Education, Universidad Viña del Mar, Viña del Mar 2520000, Chile; guillermo.cortes@uvm.cl; 8 Alicante Institute for Health and Biomedical Research (ISABIAL), 03010 Alicante, Spain

**Keywords:** spinal cord injury, tetraplegia, body composition, bioimpedance analysis, dietary intake, lipid profile, cardiometabolic risk, adaptive sports

## Abstract

*Background and Objectives*: Individuals with chronic tetraplegia frequently present altered body composition and metabolic dysregulation, which may not be adequately reflected by traditional markers such as body mass index. This study aimed to evaluate body composition, dietary patterns, and biochemical profiles in adults with chronic tetraplegia, and to explore cross-domain associations between these outcomes. *Materials and Methods*: Eleven adults with chronic tetraplegia underwent anthropometric assessment (BMI, body fat %, triceps skinfold), dietary evaluation, and fasting biochemical analysis (lipids and glucose). Data distribution was tested with Shapiro–Wilk. Between-sex comparisons were explored with Mann–Whitney U tests. Pearson correlations were performed across domains (diet—body composition; diet—biochemical markers; body composition—biochemical markers). Statistical significance was set at *p* < 0.05. *Results*: Despite normal BMI values, participants showed elevated body fat percentages. Dietary intake was characterized by excessive lipid consumption and suboptimal protein contribution. Cross-domain correlations revealed that higher energy and macronutrient intakes were associated with one another. Protein intake was inversely correlated with triglyceride levels (r = −0.63, *p =* 0.038), while triceps skinfold showed a strong correlation with body fat percentage (r = 0.78, *p* = 0.004). Fasting glucose was positively correlated with total cholesterol (r = 0.61, *p* = 0.046). Most correlations did not reach statistical significance, likely due to limited sample size, but provided exploratory insight into the interplay between diet, adiposity, and metabolic markers. *Conclusions*: Individuals with chronic tetraplegia may exhibit increased adiposity and early metabolic alterations despite normal BMI and modest reported energy intake. These findings reinforce the inadequacy of BMI for nutritional assessment in SCI and highlight the need for integrated evaluation—including body composition, dietary quality, and biochemical monitoring—to guide personalized interventions aimed at reducing cardiometabolic risk.

## 1. Introduction

Spinal cord injury (SCI) often leads to profound and chronic alterations in metabolism, body composition, and nutritional status. Individuals with tetraplegia—injuries at the cervical level—experience complete or partial loss of motor and sensory function in all four limbs and the trunk. Consequently, they typically present with considerable muscle atrophy, reduced resting metabolic rate (RMR), and diminished ambulatory activity compared with persons with paraplegia [1]. Meta-analyses show that individuals with tetraplegia have significantly higher body fat percentage (mean difference ≈1.9%) and lower lean mass (≈3.0 kg) than those with paraplegia, despite having a lower BMI [2]. Central adiposity, as measured by visceral adipose tissue, is also elevated in tetraplegia (weighted mean difference of 0.24 dm^2^), highlighting pro-inflammatory and cardiometabolic risk [2].

This condition—often referred to as “neurogenic obesity” or the “obesity hidden phenotype”—results from a positive energy balance in which caloric intake exceeds reduced expenditure. A meta-analysis including over 600 individuals with chronic SCI reported a pooled daily energy intake of approximately 1876 kcal, an average RMR of 1492 kcal, and an estimated total daily energy expenditure (TDEE) of ~1716 kcal, yielding a surplus of more than 150 kcal/day [3]. Even modest imbalances contribute over time to adipose tissue accumulation, particularly central fat, and promote insulin resistance and dyslipidemia [3].

Despite these risks, nutritional assessment and dietary management in SCI remain inconsistent. Self-reported dietary methods such as food diaries and recalls often underestimate intake, especially in individuals with tetraplegia due to functional limitations affecting accurate reporting [4,5]. Macronutrient imbalances are common: carbohydrate intake frequently exceeds recommendations (~969 kcal/day), and fat accounts for ~35% of total energy—often surpassing saturated fat limits (<10%) [3]. Protein intake typically meets or slightly exceeds general recommendations (~15–17% of energy), but qualitative differences in amino acid profiles (e.g., lower lysine or leucine content) may exist, particularly in tetraplegia [3]. Such imbalances may contribute to continued muscle wasting and fat accumulation despite apparently adequate intake.

Clinical guidelines consistently recommend comprehensive nutritional screening in SCI, including anthropometric, biochemical, and dietary assessment performed by trained dietitians [6]. However, implementation remains inconsistent, and many care settings rely mainly on basic anthropometry, which systematically underestimates adiposity in SCI [6]. Few studies have simultaneously assessed anthropometry, dietary intake, and biochemical parameters within the same sample. Existing work is predominantly descriptive or focused on educational interventions that fail to demonstrate significant improvements in metabolic health unless embedded in more comprehensive strategies [7].

Recent reviews highlight the need for multimodal assessment—including bioimpedance analysis (BIA), indirect calorimetry, and next-generation metabolomics—to improve accuracy in evaluating body composition and nutritional status in SCI [1]. While BIA is frequently used due to its accessibility and non-invasiveness [8], very few studies have combined BIA-derived body fat percentage with dietary intake and biochemical markers in individuals with tetraplegia.

There is, therefore, a clear gap: the literature lacks integrated cross-sectional analyses, even in small pilot samples, linking dietary intake, anthropometric composition, and metabolic biomarkers in tetraplegia. Understanding how habitual diet relates to body fat percentage and plasma lipid/glucose profiles—particularly in individuals with normal BMI but potentially elevated adiposity—could clarify risk trajectories and guide targeted nutritional strategies.

Integrating dietary intake, body composition, and biochemical biomarkers into a single analytical framework offers added clinical value in SCI, as each domain captures different but interrelated aspects of metabolic dysregulation [2,9,10,11]. Body composition reflects the profound muscle atrophy and neurogenic adiposity characteristic of tetraplegia; dietary intake reflects modifiable behavioral factors influencing energy balance; and biochemical markers represent downstream metabolic consequences such as dyslipidemia, impaired glucose handling, and low-grade inflammation [2,10]. When assessed independently, these components provide only partial insight. When evaluated together, they enable identification of discordant phenotypes—such as normal BMI but high fat mass with an adverse lipid profile—and allow a more accurate characterization of cardiometabolic risk [12,13,14]. Despite this clinical relevance, very few studies in tetraplegia have adopted this integrated approach.

Taken together, individuals with chronic tetraplegia exhibit profound alterations in body composition and metabolism that are not fully captured by BMI. Although previous studies have described elevated adiposity, dyslipidemia, and suboptimal dietary patterns in spinal cord injury, the evidence in tetraplegia remains fragmented. Most reports assess diet, body composition, or biochemical markers separately, while few integrate these domains within the same cohort. Moreover, cross-domain associations—linking dietary intake, body composition, and metabolic biomarkers—are rarely analyzed. When such associations are reported, results are inconsistent and frequently underpowered, limiting understanding of how habitual diet relates to adiposity and metabolic dysregulation in this population. Furthermore, while adiposity—particularly total and central fat mass—appears more strongly linked to dyslipidemia, glucose alterations, and low-grade inflammation than BMI [2,9,14], the extent to which simple, clinically feasible measures such as BIA-derived body fat percentage or single-site skinfolds reflect metabolic profiles in tetraplegia remains unclear [12,13,15,16,17].

Given this gap, there is a need for integrated analyses that simultaneously characterize dietary intake, body composition, and biochemical status in individuals with tetraplegia, to identify potential interrelationships and estimate effect sizes that can guide future adequately powered studies.

Accordingly, this cross-sectional pilot study was designed to (1) assess nutritional status through body composition (BIA and skinfold), dietary intake (energy and macronutrient distribution), and biochemical markers (total cholesterol, triglycerides, fasting glucose, liver enzymes, and creatinine); and (2) explore associations between dietary intake and biochemical markers, and between body fat percentage and lipid/glucose values. These associations were selected based on prior evidence indicating that dietary patterns influence body composition more consistently than they influence circulating metabolic biomarkers in individuals with tetraplegia [12,13,15]. In this population, profound muscle atrophy and increased adiposity arise predominantly from neurogenic mechanisms and chronic physical inactivity, making dietary effects on biochemical parameters relatively modest and inconsistent [2,16,17]. Conversely, adiposity—particularly total and central fat mass—is strongly and directly associated with dyslipidemia, impaired glucose regulation, and low-grade inflammation, and is a more accurate predictor of metabolic dysfunction than BMI [2,9,14]. Therefore, investigating diet-biochemical relationships and body fat-metabolic relationships offers clinically meaningful insight into both modifiable behaviors and structural metabolic risk factors.

## 2. Materials and Methods

### 2.1. Study Design and Participants

A cross-sectional pilot study was conducted, including 11 adults with chronic SCI. The sample consisted mainly of cervical injuries (C4–C7), although a small number of thoracic and upper lumbar injuries (T6–L1) were also represented. All participants had sustained their injury more than one year before evaluation, were clinically stable, and regularly engaged in adapted physical activity (sailing simulator).

Inclusion criteria were age ≥ 18 years, diagnosis of traumatic or non-traumatic SCI at cervical or upper thoracic levels, and ability to provide informed consent. Exclusion criteria included: acute infection; chronic inflammatory diseases unrelated to the injury (e.g., rheumatoid arthritis, inflammatory bowel disease, systemic lupus erythematosus); severe organ failure (e.g., renal or hepatic); or ongoing nutritional supplementation specifically aimed at weight loss or gain.

Notably, all participants regularly engaged in adaptive sailing simulator training as part of their physical activity routine, which predominantly involves upper-body effort and postural control.

### 2.2. Ethical Considerations

The study was approved by the Ethics Committee of the Universidad Católica San Antonio de Murcia (UCAM) (protocol code CE021912, approved on 1 February 2019) and conducted in accordance with the principles of the Declaration of Helsinki. Written informed consent was obtained from all participants prior to inclusion.

### 2.3. Anthropometric and Body Composition Assessment

Body weight was measured using a calibrated wheelchair scale. Net body weight was obtained by subtracting the previously determined weight of each participant’s wheelchair. Recumbent length was measured in the supine position using a flexible anthropometric tape, as standing height measurement was not feasible due to functional limitations. Body mass index (BMI) and body fat percentage were obtained using a bioelectrical impedance analysis (BIA) device (OMRON, Omron Healthcare Co., Ltd., Kyoto, Japan) validated for individuals with limited mobility.

The thickness of the triceps skinfold was measured using a calibrated skinfold caliper, following the standardized anthropometric procedures described by the International Society for the Advancement of Kinanthropometry (ISAK) [18]. Body fat percentage from triceps skinfold was estimated using the equation validated for individuals with spinal cord injury by Desport et al. (2000) [19]. This method is considered practical and reliable in this population, particularly when multi-site skinfold assessment is not feasible.

### 2.4. Dietary Assessment

Dietary intake was assessed using a 3–7-day weighed food diary, including both weekdays and weekend days. Participants recorded all foods and beverages consumed, specifying quantities, preparation methods, and brands. The diary was administered in person, with written instructions and examples, and monitored by a trained dietitian through telephone follow-up every 48–72 h to ensure completeness. This method has been validated in SCI populations and shows acceptable reliability for estimating habitual intake [3,20,21].

Energy intake and macronutrient distribution were analyzed using validated nutritional analysis software based on standard food composition tables. Data were expressed as total energy (kcal/day) and percentage of total energy derived from each macronutrient.

### 2.5. Biochemical Analysis

Venous blood samples were collected after a 10–12 h overnight fast. The following parameters were analyzed using automated laboratory techniques: fasting glucose, total cholesterol, triglycerides, urea, creatinine, uric acid, electrolytes (sodium, potassium, chloride, calcium, albumin-corrected calcium, and phosphorus), total proteins, albumin, liver enzymes (aspartate aminotransferase, AST; alanine aminotransferase, ALT; gamma-glutamyl transferase, GGT), bilirubin, alkaline phosphatase, and lactate dehydrogenase (LDH).

### 2.6. Statistical Analysis

All statistical analyses were performed using Jamovi (Version 2.6.17.0). Continuous variables were tested for normality using the Shapiro–Wilk test. Normally distributed variables were reported as mean ± standard deviation (SD), whereas non-normally distributed variables were expressed as median and interquartile range (IQR).

Differences between men and women were examined using the Mann–Whitney U test. Although the study was not powered for subgroup analysis, these comparisons were included to provide exploratory insight into potential sex-related variability in nutritional and metabolic status in tetraplegia.

Analyses focusing on biochemical parameters were restricted to lipid profile (total cholesterol, HDL, LDL, triglycerides) and fasting glucose. These biomarkers were selected a priori because they represent the most physiologically relevant indicators of cardiometabolic status in spinal cord injury and show the strongest documented associations with adiposity and dietary patterns in individuals with tetraplegia. Other laboratory markers (e.g., total proteins, creatinine, electrolytes) were not included in the correlation analyses because they are not consistently linked to nutritional intake or body composition in this population and, therefore, did not align with the study objectives.

Associations between body composition (% body fat), dietary intake, and biochemical markers were assessed using Pearson correlation coefficients. Effect sizes (r) were calculated and interpreted according to Cohen’s criteria. Statistical significance was set at *p* < 0.05.

## 3. Results

### 3.1. Characteristics of the Sample

The study included 11 adults with chronic SCI, of whom 7 were men and 4 were women. The mean age was 45.5 ± 13.2 years, and the mean time since injury was 5.4 ± 0.9 years. Most participants presented high-level lesions, including cervical injuries (C4–C7), while three individuals presented thoracic or upper lumbar injuries (T6, T6, L1). ASIA impairment grades ranged from A to D.

Participants had a mean height of 170.3 ± 5.1 cm and a median body weight of 63.0 kg (IQR 10.3). The mean BMI was 22.1 ± 3.0 kg/m^2^. All participants were clinically stable and engaged in regular adapted physical activity through sailing simulator training.

### 3.2. Anthropometric and Body Composition Characteristics

Table 1 presents the individual characteristics of all participants, including age, time since injury, anthropometric data, and neurological level. Table 2 summarizes the anthropometric and body composition parameters stratified by sex. Significant sex differences were observed in height, body fat percentage, and triceps skinfold thickness (*p* < 0.05), with women showing higher adiposity markers and men being taller. No significant differences were found in body weight. Effect sizes were large for skinfold and body fat, supporting a clear sex-related pattern in fat distribution.

### 3.3. Dietary Intake

Table 3 displays the energy and macronutrient intake of the participants. The average daily energy intake was approximately 1960 kcal, with mean protein, carbohydrate, and lipid intake values close to 94 g, 189 g, and 91 g/day, respectively. No significant differences were found between women and men in any of the dietary variables (*p* > 0.78), and effect sizes were small to negligible, indicating similar dietary patterns across sexes.

### 3.4. Biochemical Parameters

Table 4 shows the biochemical profile of the participants. No statistically significant sex differences were observed in glucose, triglycerides, renal function markers, or most electrolytes and minerals. Although women presented higher total cholesterol levels and men showed higher creatinine and uric acid, these differences did not reach statistical significance.

### 3.5. Correlations

The full correlation matrix between dietary intake, body composition and biochemical markers is presented in Table 5. Analysis of cross-domain correlations (diet–body composition–biomarkers) revealed several noteworthy associations. Higher energy intake was strongly correlated with protein (r = 0.66, *p =* 0.027), carbohydrate (r = 0.80, *p =* 0.003) and lipid intake (r = 0.81, *p =* 0.002), indicating that macronutrients increased proportionally with total calories. Percentage body fat showed a strong positive correlation with triceps skinfold thickness (r = 0.78, *p =* 0.004), supporting the validity of this single-site skinfold as a proxy for adiposity in this group. Regarding diet-biomarker relationships, higher protein intake was inversely associated with triglyceride levels (r = −0.63, *p =* 0.038). In addition, fasting glucose was positively correlated with total cholesterol (r = 0.61, *p =* 0.046).

## 4. Discussion

The present study provides a multidimensional analysis of body composition, dietary intake, and biochemical markers in individuals with chronic tetraplegia, revealing elevated adiposity and suboptimal dietary patterns despite normal BMI values. These findings reinforce the limitations of BMI as a diagnostic tool for adiposity in this population, a fact consistently reported in previous research, particularly in cervical lesions [14,22].

In a large cross-sectional study, Spungen et al. reported significantly higher fat mass in tetraplegia compared with able-bodied controls of similar BMI, highlighting the phenomenon of “hidden obesity” [22]. Nightingale et al. (2017) [23] further documented increased visceral adipose tissue and reduced appendicular lean mass in tetraplegia, independent of BMI. The use of bioelectrical impedance analysis (BIA) and triceps skinfold–derived estimates, as applied in this study, provides a feasible approach in clinical practice, although DXA remains the gold standard for body composition analysis [24].

The dietary profile identified, characterized by excessive fat intake and suboptimal protein contribution, is consistent with prior findings in SCI populations. Farkas et al. (2019) [3] reported that fat intake often exceeds 35% of total energy, with saturated fat surpassing recommended thresholds, and is associated with central adiposity and dyslipidemia. Pelletier et al. (2014) [25] observed that although protein intake in SCI patients may meet general population recommendations, it is frequently insufficient to counteract anabolic resistance and progressive muscle atrophy in tetraplegia. These observations indicate that current nutritional guidelines may require specific adaptations for SCI, focusing on protein quality and lipid profile optimization.

The observed correlations between higher body fat percentage and adverse lipid profiles—particularly elevated triglycerides—are in line with previous evidence [26,27]. Such alterations can manifest even with modest energy intakes, reflecting reduced total daily energy expenditure and qualitative imbalances in macronutrient distribution [28]. High lipid consumption, coupled with inadequate protein intake, may aggravate insulin resistance and low-grade systemic inflammation, further deteriorating metabolic health [24].

Several underlying mechanisms explain these findings. Loss of skeletal muscle mass significantly reduces resting metabolic rate, while physical inactivity limits lipid oxidation and glucose uptake [29]. Increased visceral adiposity contributes to chronic low-grade inflammation, which promotes atherogenic dyslipidemia [30]. Inadequate protein intake, superimposed on anabolic resistance, accelerates muscle loss, indirectly facilitating fat accumulation.

Beyond within-domain associations, the cross-domain correlations provide additional insight into how dietary intake, body composition and biochemical markers interact in chronic tetraplegia. Higher protein intake was inversely associated with triglyceride levels, suggesting a potential protective effect of adequate protein consumption on lipid metabolism in this population. Fasting glucose was positively correlated with total cholesterol, consistent with early clustering of metabolic risk factors previously described in individuals with SCI. By contrast, dietary variables did not correlate with percentage body fat or triceps skinfold, and no significant associations were observed between body composition indicators and biochemical markers. These findings may reflect the small sample size, the relatively homogeneous dietary patterns of the cohort, and the attenuated variability in metabolic biomarkers typically observed in physically active individuals with SCI. Nonetheless, the direction of the associations aligns with established metabolic alterations in neurogenic obesity and highlights the need for larger studies to clarify these interrelationships.

In this cohort, all participants regularly engaged in adaptive sailing simulator training, a modality emphasizing upper-body movement and postural control. Although this activity was not quantified or analyzed in relation to nutritional or metabolic outcomes, its inclusion provides context regarding the physical activity habits of the sample. Previous literature indicates that upper-body aerobic and resistance exercise can improve cardiorespiratory fitness and muscular strength in SCI [31,32,33], although evidence for changes in body composition remains inconsistent [31,33,34,35,36]. Therefore, while physical activity may contribute to the overall health profile of participants, no conclusions can be drawn regarding its influence on the patterns observed in this study.

An important strength of this study lies in its integration of anthropometric, dietary, and biochemical evaluations within the same cohort, allowing for a comprehensive overview of nutritional and metabolic status. Nonetheless, certain limitations must be acknowledged. The main limitation of this study is its small sample size, inherent to its pilot design, which restricts generalizability and limits statistical power to detect small-to-moderate correlations. As a result, non-significant findings should be interpreted with caution, as they may reflect insufficient power rather than true absence of associations. BIA and single-site skinfold measurements, while validated for SCI, may underestimate or overestimate body fat compared with multi-compartment models. Dietary assessment relied on self-report, with potential underreporting bias. The cross-sectional design precludes establishing causal relationships between dietary patterns, body composition, and biochemical parameters. Potential confounding factors such as age, sex, time since injury, and habitual physical activity were not adjusted for in the analysis, which may have influenced the observed associations. Finally, all participants were recreational athletes regularly engaged in adapted sports, which may limit external validity. Their higher-than-average physical activity levels may attenuate or modify diet–metabolism relationships compared with less active individuals with tetraplegia. Future studies with larger samples are needed to confirm and expand upon these exploratory results.

Clinically, the results support the need for routine, comprehensive nutritional assessments in SCI, incorporating body composition analysis, dietary evaluation, and biochemical screening. Interventions emphasizing high-quality protein sources, reduced saturated fat intake, and regular metabolic monitoring could help mitigate adiposity and reduce cardiovascular risk.

These findings highlight several avenues for future investigation. Larger, multi-center studies should confirm the observed associations and evaluate their progression over time. Longitudinal and interventional trials are needed to determine whether targeted nutritional strategies—such as increased protein intake, lipid profile optimization, and energy balance management—can improve body composition and metabolic health in chronic tetraplegia. Moreover, the integration of physical activity programs adapted to functional capacity, combined with precision nutrition approaches, may provide synergistic benefits in reducing cardiometabolic risk.

## 5. Conclusions

Individuals with chronic tetraplegia may present elevated body fat percentage, adverse biochemical profiles and suboptimal dietary patterns despite having a normal BMI. These findings reinforce the limited value of BMI as a standalone metric in SCI and highlight the importance of integrating body composition analysis, dietary assessment and biochemical screening in routine clinical practice.

The exploratory correlations identified between dietary intake, adiposity and metabolic markers further support the interconnected nature of nutritional and metabolic health in tetraplegia, although larger studies are needed to confirm these associations. Taken together, the results underscore the need for personalized nutritional strategies—particularly regarding protein quality and lipid intake—along with regular monitoring of metabolic risk factors.

This pilot work provides preliminary insights that may guide future longitudinal and interventional research aimed at improving cardiometabolic health in individuals living with chronic tetraplegia.

## Figures and Tables

**Table 1:** Individual Characteristics of Participants (n = 11).

Participant	Age (Years)	Time Since Injury (Years)	Height (cm)	Weight (kg)	BMI (kg/m^2^)	Sex	Neurological Level
1	31	5	172	63	21.30	M	T11–A
2	51	5	167	75.7	27.14	F	L2–C
3	42	5	173	63	21.05	M	C6–B
4	45	5	153	59.9	25.59	F	T9–D
5	52	3	167	61.5	22.05	F	T6–A
6	31	4	165	66.6	24.46	F	L3–D
7	54	7	170	75	25.95	M	T6–C
8	46	3	175	71	23.18	M	T3–A
9	29	6	190	62	17.17	M	T12–C
10	25	7	169	74	25.91	M	T6–A
11	29	4	175	70	22.86	M	T6–A

BMI = body mass index; C = cervical; F = female; kg = kilograms; L = lumbar; M = male; T = thoracic; The letter next to the height of the injury corresponds to the ASIA scale (American Spinal Injury Association Impairment Scale).

**Table 2:** Anthropometric and body composition characteristics of the sample.

	Total (n = 11)		Women (n = 4)		Men (n = 7)		*p*	Mean Difference	Effect Size
	Mean	SD	Mean	SD	Mean	SD			
Height (m)	1.71	0.09	1.63	0.07	1.75	0.07	0.010	−0.080	10.00
Body fat (%)	22.3	10.80	34.4	3.60	15.5	5.93	0.010	196.259	−10.00
Triceps SF (mm)	16.7	6.48	23.1	2.25	13.0	4.90	0.011	89.151	−10.00
	**Median**	**IQR**	**Median**	**IQR**	**Median**	**IQR**			
Weight (kg)	63.0	10.30	64.0	7.77	63.0	9.50	0.776	−0.913	0.142

kg = kilograms; m = meters; % = percentage; mm = millimeters; SF = skinfold. Data are expressed as mean ± standard deviation (SD) and interquartile range (IQR). *p*-values correspond to Mann–Whitney U tests comparing men and women.

**Table 3:** Dietary intake (energy and macronutrients).

	Total (n = 11)		Women (n = 4)		Men (n = 7)		*p*	Mean Difference	Effect Size
	Mean	SD	Mean	SD	Mean	SD			
Energy intake (kcal)	1963	309	1991	248	1947	358	0.788	516.000	−0.1429
Protein intake (g/day)	93.8	20.0	97.5	21.7	91.6	20.4	1.000	0.9750	0.0000
CH intake (g/day)	189.0	42.2	188.0	27.5	189.0	50.9	0.927	78.125	−0.0714
Lipid intake (g/day)	91.0	14.8	93.1	21.6	89.9	11.3	0.788	60.000	−0.1429

kcal = kilocalories; g = grams; CH = carbohydrate. Data are expressed as mean ± standard deviation (SD) and interquartile range (IQR). *p*-values correspond to Mann–Whitney U tests comparing men and women.

**Table 4:** Biochemical parameters (metabolic profile, renal and liver function).

	Total (n = 11)		Women (n = 4)		Men (n = 7)		*p*	Mean Difference	Effect Size
	Mean	SD	Mean	SD	Mean	SD			
Metabolic profile									
Total, cholesterol (mg/dL)	173.0	29.8	192.0	17.7	162.0	30.8	0.230	297.500	−0.5000
Triglycerides (mg/dL)	76.7	15.4	82.5	17.0	73.5	14.7	0.527	93.000	−0.2857
Renal function									
Urea (mg/dL)	34.5	10.6	37.5	14.4	32.9	8.7	0.705	21.933	−0.1786
Creatinine (mg/dL)	0.62	0.11	0.61	0.15	0.62	0.09	0.788	0.040	−0.1429
Acid Uric (mg/dL)	4.74	1.44	4.45	1.32	4.90	1.58	0.412	−0.600	0.3571
Electrolytes and minerals									
Sodium (mmol/L)	141.0	2.32	141.0	3.00	141.0	2.11	1.000	0.400	0.0000
Potassium (mmol/L)	4.01	0.33	3.95	0.32	4.04	0.36	0.648	−0.115	0.2143
Chloride (mmol/L)	102	2.27	102	3.19	102	1.87	0.504	−0.500	0.2857
Calcium (mg/dL)	9.23	0.39	9.22	0.46	9.23	0.38	1.000	0.083	−0.0357
Albumin-corrected calcium (mg/dL)	9.18	0.19	9.20	0.18	9.17	0.21	0.847	4.19 × 10^−5^	−0.1071
Phosphorus (mg/dL)	4.05	0.29	3.85	0.19	4.17	0.29	0.103	−0.319	0.6429
Protein status									
Total, proteins (g/dL)	6.54	0.61	6.36	0.41	6.64	0.70	0.315	−0.330	0.4286
Albumin (g/dL)	4.15	0.28	4.05	0.37	4.21	0.23	0.563	−0.099	0.2500
Liver function and enzymes									
ALT (U/L)	26.8	13.2	25.5	20.4	27.6	9.1	0.507	−93.000	0.2857
GGT (U/L)	10.9	3.00	11.3	4.35	10.7	2.32	0.527	19.000	−0.2857
Bilirubin (mg/dL)	0.69	0.39	0.41	0.17	0.84	0.40	0.107	−0.399	0.6429
Alkaline phosphatase (U/L)	74.0	19.10	57.3	9.64	83.6	16.30	0.029	−269.756	0.8571
LDH (U/L)	346	80.0	303	53.6	371	85.3	0.164	−505.000	0.5714
	**Median**	**IQR**	**Median**	**IQR**	**Median**	**IQR**			
Glucose (mg/dL)	84	5.5	83	2.0	85	6.5	0.505	−19.999	0.2857
AST (U/L)	22.1	12.1	17.5	8.5	22.3	9.5	0.218	−42.000	0.5000

AST = aspartate aminotransferase; ALT = alanine aminotransferase; GGT = gamma-glutamyl transferase; LDH = lactate dehydrogenase. Data are expressed as mean ± standard deviation (SD) and interquartile range (IQR).

**Table 5:** Pearson correlations between dietary intake, body composition, and biochemical markers.

	Energy (kcal)	Protein	CHO	Lipid	Body Fat (%)	Triceps SF	Cholesterol	Triglycerides	Glucose
Protein	0.66 * (0.027)	—	—	—	—	—	—	—	—
CHO	0.80 ** (0.003)	0.22 (0.518)	—	—	—	—	—	—	—
Lipid	0.81 ** (0.002)	0.70 * (0.016)	0.36 (0.278)	—	—	—	—	—	—
Body fat (%)	0.14 (0.684)	0.44 (0.177)	−0.07 (0.847)	0.22 (0.524)	—	—	—	—	—
Triceps SF	0.00 (0.997)	0.32 (0.340)	−0.09 (0.800)	0.03 (0.931)	0.78 * (0.004)	—	—	—	—
Cholesterol	0.10 (0.770)	0.39 (0.241)	−0.10 (0.774)	0.18 (0.591)	0.60 (0.052)	0.54 (0.084)	—	—	—
Triglycerides	−0.58 (0.059)	−0.63 * (0.038)	−0.35 (0.294)	−0.52 (0.104)	0.32 (0.332)	0.32 (0.332)	0.10 (0.767)	—	—
Glucose	0.43 (0.189)	0.34 (0.302)	0.41 (0.207)	0.20 (0.520)	−0.09 (0.796)	0.56 (0.046)	0.61 * (0.046)	0.20 (0.553)	—

Values represent Pearson’s correlation coefficients (r), with *p*-values shown in parentheses. * *p* < 0.05; ** *p* < 0.001. CHO: carbohydrate; SF: skinfold.

## Data Availability

Data presented in this study are available upon request from the corresponding author. The data are not publicly available due to its personal health information.

## Abbreviations

The following abbreviations are used in this manuscript:

SCI	Spinal Cord Injury
TDEE	Estimated total daily energy expenditure
BMI	Body Mass Index
BIA	Bioelectrical Impedance Analysis
DXA	Dual-energy X-ray Absorptiometry
HDL	High-Density Lipoprotein
SD	Standard Deviation
IQR	Interquartile Range
CH	Carbohydrate
RMR	Resting Metabolic Rate
ALT	Alanine Aminotransferase
AST	Aspartate Aminotransferase
GGT	Gamma-Glutamyl Transferase
WC	Waist Circumference
HC	Hip Circumference
LDH	Lactate dehydrogenase
SF	Skinfold
ISAK	Society for the Advancement of Kinanthropometry
